# Nitrogen and phosphorus uptake rates of different species from a coral reef community after a nutrient pulse

**DOI:** 10.1038/srep28821

**Published:** 2016-06-29

**Authors:** Joost den Haan, Jef Huisman, Hannah J. Brocke, Henry Goehlich, Kelly R. W. Latijnhouwers, Seth van Heeringen, Saskia A. S. Honcoop, Tanja E. Bleyenberg, Stefan Schouten, Chiara Cerli, Leo Hoitinga, Mark J. A. Vermeij, Petra M. Visser

**Affiliations:** 1 Department of Aquatic Microbiology, Institute for Biodiversity and Ecosystem Dynamics, University of Amsterdam, PO Box 94248, 1090 GE Amsterdam, The Netherlands; 2 Department of Biogeochemistry, Max Planck Institute for Marine Microbiology, Celsiusstraße 1, D-28359 Bremen, Germany; 3 Leibniz Center for Tropical Marine Ecology, Fahrenheitstraße 6, D-28359 Bremen, Germany; 4 University of Rostock, Albert-Einstein-Straße 3, 18059 Rostock, Germany; 5 NIOZ Royal Netherlands Institute for Sea Research, Department of Marine Microbiology and Biogeochemistry, and Utrecht University, P.O. Box 59, 1790 AB Den Burg, Texel, The Netherlands; 6 Department of Earth Surface Science, Institute for Biodiversity and Ecosystem Dynamics, University of Amsterdam, PO Box 94248, 1090 GE Amsterdam, The Netherlands; 7 CARMABI Foundation, Piscaderabaai z/n, PO Box 2090, Willemstad, Curaçao

## Abstract

Terrestrial runoff after heavy rainfall can increase nutrient concentrations in waters overlying coral reefs that otherwise experience low nutrient levels. Field measurements during a runoff event showed a sharp increase in nitrate (75-fold), phosphate (31-fold) and ammonium concentrations (3-fold) in waters overlying a fringing reef at the island of Curaçao (Southern Caribbean). To understand how benthic reef organisms make use of such nutrient pulses, we determined ammonium, nitrate and phosphate uptake rates for one abundant coral species, turf algae, six macroalgal and two benthic cyanobacterial species in a series of laboratory experiments. Nutrient uptake rates differed among benthic functional groups. The filamentous macroalga *Cladophora* spp., turf algae and the benthic cyanobacterium *Lyngbya majuscula* had the highest uptake rates per unit biomass, whereas the coral *Madracis mirabilis* had the lowest. Combining nutrient uptake rates with the standing biomass of each functional group on the reef, we estimated that the ammonium and phosphate delivered during runoff events is mostly taken up by turf algae and the two macroalgae *Lobophora variegata* and *Dictyota pulchella*. Our results support the often proposed, but rarely tested, assumption that turf algae and opportunistic macroalgae primarily benefit from episodic inputs of nutrients to coral reefs.

Discharge of domestic and industrial sewage, terrestrial runoff from urbanized and agricultural land and aquaculture effluent have all contributed to a greater availability of nutrients such as nitrogen and phosphorus on coral reefs worldwide^1^,^2^,^3^. Increased nutrient availability alleviates nutrient limitation of benthic algae and cyanobacteria^4^,^5^,^6^, providing opportunistic species with resources needed for growth and reproduction. This allows these species to occupy or overtake space on reefs at the expense of live corals^1^,^7^,^8^,^9^,^10^,^11^,^12^,^13^,^14^,^15^,^16^,^17^,^18^,^19^,^20^, especially when levels of herbivory are low^8^.

The consequences of coastal eutrophication for benthic macroalgal communities have been studied extensively in temperate coastal waters^9^,^10^,^11^,^12^. Increased nitrogen and phosphorus concentrations in these waters often resulted in large-scale macroalgal blooms, especially of species belonging to the genera *Enteromorpha*, *Ulva*, *Cladophora*, and *Chaetomorpha*^13^,^14^. These macroalgal species are capable of quickly capitalizing on the high availability of nutrients, even when high nutrient levels last only for a short period of time (i.e., minutes to hours)^10^,^14^,^15^. The proliferation of macroalgae stimulated by an episodic increase in nutrient availability alters the benthic community structure of coastal ecosystems for long periods thereafter^13^.

In contrast to temperate coastal waters, episodic increases in nutrient availability have received less attention for waters overlying coral reefs. Concentrations of dissolved inorganic nitrogen (DIN) and phosphorus (DIP) in reef waters are often assumed to be low (i.e., several micromoles of DIN and tenths of micromoles of DIP) and relatively constant throughout the year^16^,^17^. Water sampling efforts are therefore often conducted on relatively coarse temporal and spatial sampling scales (i.e., weeks to years at widely separated sites and depths)^18^, whereby episodic runoff events that last only a few hours likely remain unrecorded. Only few studies have quantified the duration and magnitude of nutrient increases during eutrophication events on coral reefs (e.g. prolonged sewage discharge)^21^,^22^,^23^.

Coral reefs around the island of Curaçao experience episodic runoff events after heavy rainfall in the form of sediment plumes composed of terrestrial runoff that are clearly visible above (Fig. 1a,b) and below the water surface (Fig. 1c,d). Sediment plumes can elevate local DIN and DIP concentrations in the water column substantially (up to 400 times)^2^. Recently, we showed that growth of the macroalga *Lobophora variegata* on the fringing reefs of Curacao was co-limited by nitrogen and phosphorus^6^, indicating that elevated DIN and DIP concentrations potentially may have major effects on these ecosystems. Not much is known, however, about the nutrient uptake rates of macroalgae and other species inhabiting the coral reefs when exposed to temporarily elevated nutrient levels. To assess if certain benthic functional groups take up more nutrients than others during episodic eutrophication events, we determined the nitrogen and phosphorus uptake rates of corals, macroalgae, benthic cyanobacteria, and turf algae during a simulated nutrient pulse in a series of laboratory experiments. The nutrient concentrations in this nutrient pulse were based on concentrations used in previous eutrophication experiments on reefs^20^,^24^,^25^,^26^, and turned out to be comparable to the concentrations measured in a sediment plume in the field during a runoff event on November 23^rd^, 2011 (shown in Fig. 1). This enabled us to assess if episodic nutrient inputs resulting from rain-induced sediment plumes may benefit certain benthic species more than others, potentially altering the competitive relationships among them.

## Results

### Nutrient enrichment after rainfall on the reef

**Figure 1:**
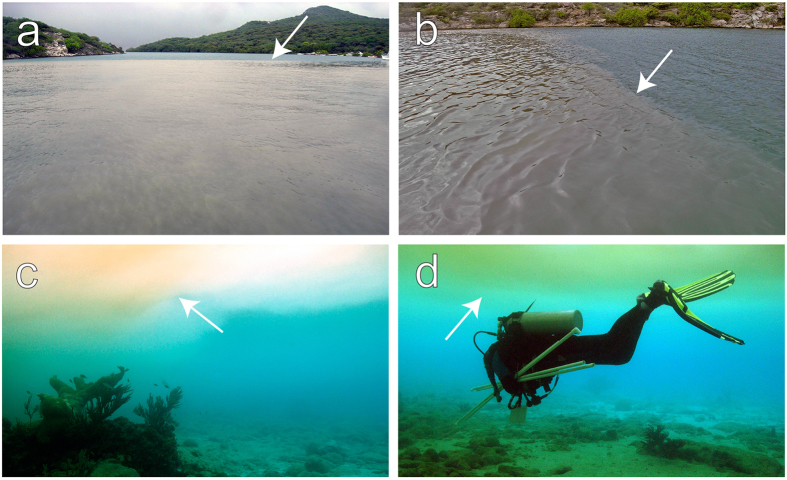
Episodic runoff events. (**a**,**b**) Sediment plume at the outlet of Piscadera Bay on 23 November 2011 after a period of heavy rainfall; arrows indicate the front between the sediment plume and clear oceanic water. The sediment plume lasted <1 hr. (**c**,**d**) Underwater view of a sediment plume extending from the water surface to ~3 m depth at the dive site ‘Pest Bay’ (12°9′59.77″N, 69°0′43.91″W) on 21 October 2010. Image credit: Hannah J. Brocke and Joost den Haan.

**Figure 2:**
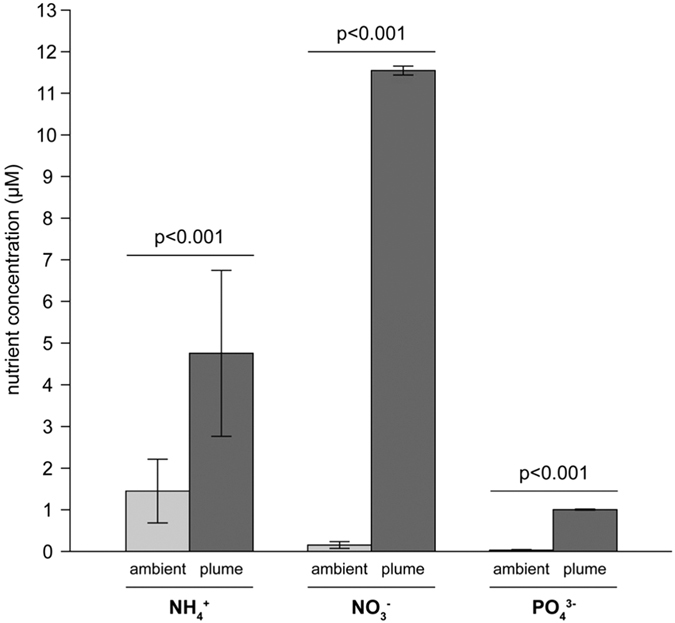
Nutrient enrichment after heavy rainfall. Ambient nutrient concentrations at Buoy 0 sampled between 31 October–10 December 2011 (light grey bars) are compared against the nutrient concentrations in the sediment plume observed at the outlet of Piscadera Bay on 23 November 2011 (dark grey bars). Error bars represent s.d. of the mean (n = 30 for ambient nutrient concentrations and n = 6 for sediment plume). Differences between the ambient nutrient concentrations and those in the sediment plume were tested by the two-sample Student’s *t*-test (for equal variances) or the Welch’s *t*-test (for unequal variances).

Ambient nutrient concentrations (mean ± s.e.m.) above the studied reef were low: 1.45 ± 0.14 μM for NH_4_^+^, 0.15 ± 0.02 μM for NO_3_^−^ and 0.032 ± 0.003 μM for PO_4_^3−^. We sampled a sediment plume at the outlet of the nearby Piscadera Bay on 23 November 2011 after a period of heavy rainfall (Fig. 1a,b). Seawater collected from the sediment plume was characterized by 75-fold higher NO_3_^−^ concentrations (Welch’s *t*-test: *t* = −36.1, df = 29.1, *P* < 0.001), 31-fold higher PO_4_^3−^ concentrations (Welch’s *t*-test: *t* = −44.6, df = 28.3, *P* < 0.001), and 3-fold higher NH_4_^+^ concentrations (Student’s *t*-test: *t* = −5.2, df = 33, *P* < 0.001) compared to ambient concentrations (Fig. 2). The N:P ratio of dissolved inorganic nutrients in seawater was nearly 50:1 under ambient conditions, whereas the N:P ratio in the sediment plume approached the Redfield ratio of 16:1. During our study period from March to June (2012 and 2013), similar sediment plumes were observed at least once per month near Buoy 0, and more frequently during the wet season (October-February). The plumes varied in size, but were usually tens of meters across and lasted from 30 min to a few hours at most.

### Nutrient uptake kinetics based on laboratory experiments

**Figure 3:**
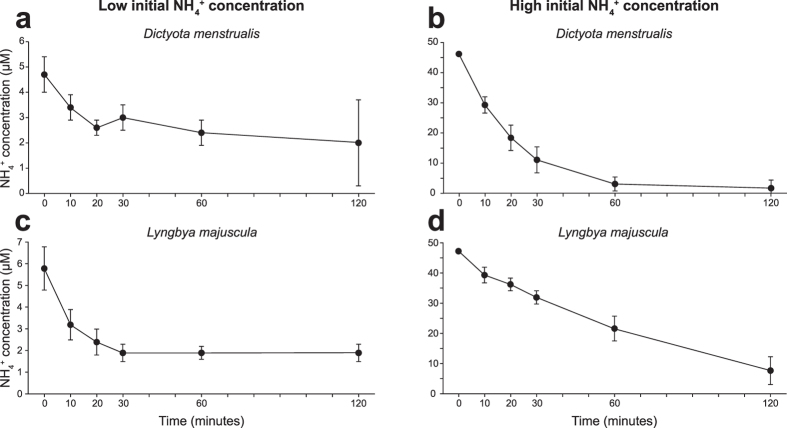
Examples of NH_4_^+^ uptake experiments in the laboratory. Decrease of NH_4_^+^ concentrations in the incubation water due to NH_4_^+^ uptake by (**a**,**b**) the macroalga *Dictyota menstrualis* (from 5 m depth) and (**c**,**d**) the benthic cyanobacterium *Lyngbya majuscula* (from 20 m depth). The species were incubated at low (left column) and at high (right column) initial NH_4_^+^ concentrations. Error bars represent s.d. of the mean (n = 9). NH_4_^+^ uptake experiments of the other species are shown in Supplementary Figs S1 and S2.

**Figure 4:**
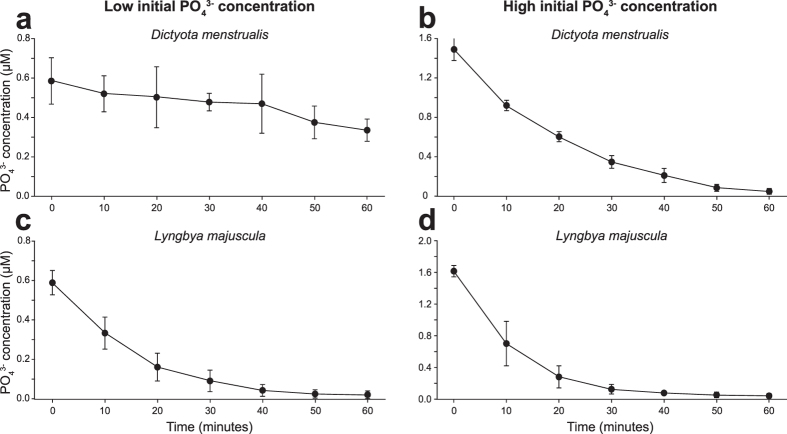
Examples of PO_4_^3−^ uptake experiments in the laboratory. Decrease of PO_4_^3−^ concentrations in the incubation water due to PO_4_^3−^ uptake by (**a**,**b**) the macroalga *Dictyota menstrualis* (from 5 m depth) and (**c**,**d**) the benthic cyanobacterium *Lyngbya majuscula* (from 20 m depth). The species were incubated at low (left column) and at high (right column) initial PO_4_^3−^ concentrations. Error bars represent s.d. of the mean (n = 9). PO_4_^3−^ uptake experiments of the other species are shown in Supplementary Figs S3 and S4.

During the nutrient uptake experiments, NH_4_^+^ and PO_4_^3−^ concentrations in the incubation water decreased (see Figs 3 and 4 and Supplementary Figs S1–S4). Uptake rates of NH_4_^+^ and PO_4_^3−^ gradually decreased as external nutrient concentrations lowered. In general, NH_4_^+^ and PO_4_^3−^ were not completely exhausted over the 2-hr incubation period, yielding residual concentrations of 1.5–8 μM NH_4_^+^ and 0.03–0.5 μM PO_4_^3−^ depending on the species that was incubated (Figs 3 and 4 and Supplementary Figs S1–S4).

**Figure 5:**
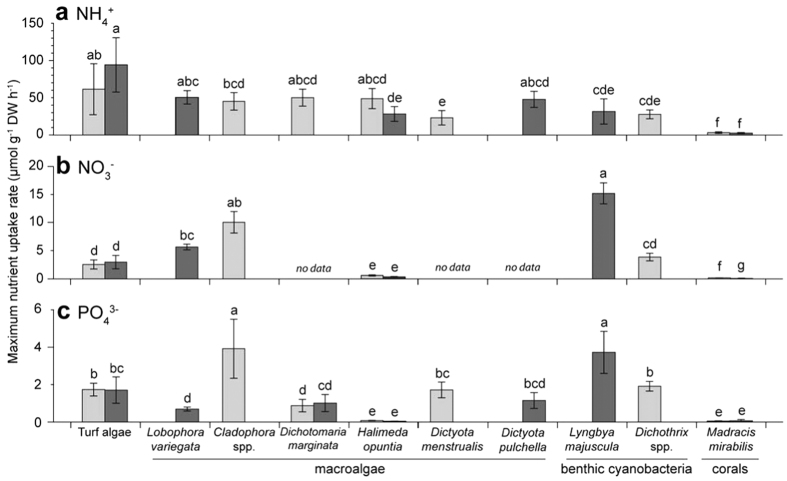
Comparison of maximum nutrient uptake rates (V_max_) of the different species. Maximum uptake rates of (**a**) NH_4_^+^, (**b**) NO_3_^−^ and (**c**) PO_4_^3−^. Shading of the bars indicates whether specimens were collected from 5 m depth (light grey bars) or 20 m depth (dark grey bars); note that not all species were present at both depths. Error bars represent s.d. of the mean (n = 9). Within each panel, bars that do not share the same letter are significantly different, as tested by two-way analysis of variance (Table 1) followed by post hoc comparison of the means.

The maximum nutrient uptake rates (V_max_) differed significantly among species (Table 1). V_max_ for NH_4_^+^ was highest for turf algae, followed by the macroalgal species and cyanobacteria, and lowest for the coral *Madracis mirabilis* (Fig. 5a). The V_max_ for NO_3_^−^ was highest for the benthic cyanobacterium *Lyngbya majuscula* and the green macroalgal genus *Cladophora*, and again lowest for *M. mirabilis* (Fig. 5b). The V_max_ for PO_4_^3−^ was again highest for *L. majuscula* and *Cladophora* spp., and lowest for *Halimeda opuntia* and *M. mirabilis* (Fig. 5c). Furthermore, we found a significant effect of depth on the V_max_ for NO_3_^−^ and PO_4_^3−^ and a significant interaction effect of species × depth on the V_max_ for all three nutrients (*P* < 0.05, see Table 1). However, post hoc comparison of the means revealed that the influence of depth on V_max_ was driven by differences in the NO_3_^−^ uptake by *M. mirabilis* between depths. V_max_ was not significantly different between depths for all other species that occurred at both depths (Fig. 5).

**Figure 6:**
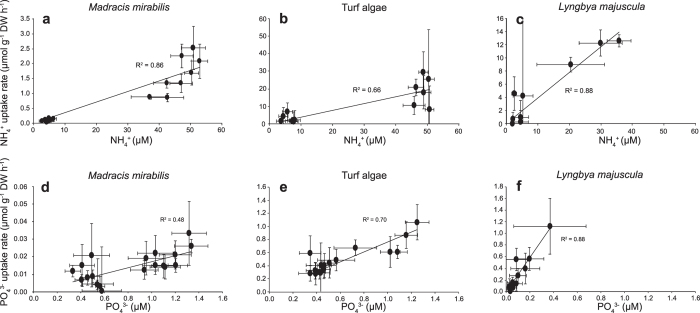
Examples of NH_4_^+^ (**a–c**) and PO_4_^3−^ (**d–f**) uptake rates of different species as a function of the external NH_4_^+^ and PO_4_^3−^ concentration. Species included are (**a,d**) the coral *Madracis mirabilis*, (**b,e**) turf algae, and (**c,f**) the cyanobacterium *Lyngbya majuscula*. NH_4_^+^ and PO_4_^3−^ uptake rates were calculated over 20 min time intervals during the nutrient uptake experiments. For turf algae and *L. majuscula*, the first 10 min of uptake was not included as they had a biphasic NH_4_^+^ and PO_4_^3−^ uptake. Data included specimens collected from both 5 and 20 m depth, and from uptake experiments performed at both low and high initial NH_4_^+^ and PO_4_^3−^ concentrations. Trend lines are based on linear regression (*P* < 0.05) forced through the origin. Error bars represent s.d. of the mean (n = 9). Results from the other species can be found in the Supplementary Figs S5 and S6.

Except for *M. mirabilis* and *Dichotomaria marginata*, the uptake of NH_4_^+^ was significantly higher during the first 10 min of the experiments than during the subsequent time intervals (see Supplementary Table S1), a pattern generally referred to as “biphasic uptake”. Surge uptake of PO_4_^3−^ was only observed in turf algae and the benthic cyanobacteria *Dichothrix* spp. and *L. majuscula* (see Supplementary Table S1). Linear regression showed that, after initial surge uptake, the NH_4_^+^ and PO_4_^3−^ uptake rates of most species were positively linearly related to the ambient NH_4_^+^ and PO_4_^3−^ concentrations, respectively (Fig. 6 and Supplementary Figs S5 and S6).

### Distribution of nutrients among different species

Since we were not able to obtain concentration-dependent uptake rates for NO_3_^−^, we focus on NH_4_^+^ and PO_4_^3−^ uptake only. The NH_4_^+^ and PO_4_^3−^ uptake rates were combined with the standing biomass of the benthic species at our study site (see Supplementary Table S2) to calculate how nutrients would be distributed over the different species after the occurrence of a run-off event on Curaçao. Because uptake rates were comparable between 5 and 20 m depth for all species except *M. mirabilis*, we assumed that nutrient uptake kinetics were similar across this depth range (Fig. 5). After 10 min of initial surge uptake, nutrient uptake rates of most species depended linearly on ambient nutrient concentrations (Fig. 6 and Supplementary Figs S5 and S6), which justifies the use of equation (6) in the Methods section to calculate the distribution of NH_4_^+^ and PO_4_^3−^ among the species (for more details, see the Methods section).

**Figure 7:**
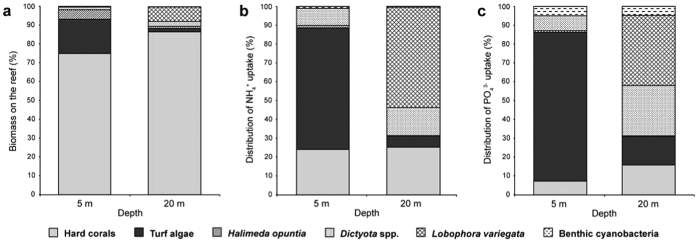
Distribution of nutrient uptake within the benthic community. (**a**) Biomass composition of the benthic community. (**b**) Distribution of total NH_4_^+^ uptake and (**c**) total PO_4_^3−^ uptake from sediment plumes over the different species and functional groups in the benthic community. The graphs in (**b**,**c**) are based on a simple model that calculates the nutrient distribution from the measured nutrient uptake kinetics and standing biomass on the reef. *Dictyota* spp. is *D. menstrualis* at 5 m and *D. pulchella* at 20 m water depth.

While corals accounted for 74.9% of the community’s total biomass at 5 m depth (Fig. 7a), according to these model calculations they took up only 24.1% of the total NH_4_^+^ and 7.3% of the total PO_4_^3−^ from the sediment plume (Fig. 7b,c). In contrast, turf algae accounted for only 18.3% of the total biomass (Fig. 7a), but took up the majority of the NH_4_^+^ and PO_4_^3−^ (64.5 and 79.0%, respectively) from the sediment plume (Fig. 7b,c). At 20 m depth, coral biomass was slightly higher (86.6%; Fig. 7a), but corals again obtained only a relatively small fraction of the total NH_4_^+^ and PO_4_^3−^ (25.3% and 15.8%, respectively; Fig. 7b,c). At 20 m, turf algae were less abundant and the brown macroalgae *Lobophora variegata* and *Dictyota pulchella* took up the majority of the available NH_4_^+^ (53.2% and 14.8%, respectively) and PO_4_^3−^ (37.2% and 26.6%, respectively) (Fig. 7b,c).

## Discussion

Our results show that sediment plumes generated by rainfall events transport highly elevated nitrate, ammonium and phosphate concentrations from the coast to near shore fringing reefs. In a series of laboratory experiments combined with a simple model, we found that such high but episodic nutrient inputs are differentially distributed among primary producers on the reef, because of species-specific differences in nutrient uptake rates and abundance. The coral *M. mirabilis* had the lowest nutrient uptake rates of all species investigated here (Fig. 5). Because we investigated only a single coral species, the results should be interpreted with some caution. However, the low uptake rates of *M. mirabilis* could be explained by the ability of corals to supplement their nutrient intake with heterotrophic feeding^27^, as direct uptake of inorganic nutrients by zooxanthellae is generally insufficient to meet corals’ nutritional demands^27^,^28^. *M. mirabilis* is indeed known for its high zooplankton capture rate^29^. We estimated that *M. mirabilis* has a V_max_ for NH_4_^+^ of ~3 μmol g^−1^ DW h^−1^ (Fig. 5a). Assuming an areal density of 0.3 g DW cm^−2^, this is equivalent to an NH_4_^+^ uptake rate of 0.21 μg N cm^−2^ min^−1^. This value is within the range of NH_4_^+^ uptake rates of 0.1–0.38 μg N cm^−2^ min^−1^ estimated for the corals *Acropora palifera*, *A. pulchra* and *Pocillopora damicornis* in the ENCORE study^20^ at the Great Barrier Reef. The NH_4_^+^ uptake rate of the red macroalga *Laurencia intricata* in the ENCORE study was an order of magnitude higher^20^. In total, these data indicate that coral species tend to have relatively low inorganic nutrient uptake rates in comparison to fast-growing species of macroalgae and turf algae.

Macroalgae, turf algae and benthic cyanobacteria acquire nitrogen and phosphorus through diffusion and/or active transport^4^,^5^,^7^. A filamentous morphology increases an alga’s surface:volume ratio, which improves its capacity to take up nutrients from its surroundings^30^,^31^. Our results confirm that species with high surface:volume ratios are characterized by high nutrient uptake rates per unit biomass. Filamentous turf algae had the highest NH_4_^+^ uptake rate, and the filamentous macroalga *Cladophora* spp. and the filamentous cyanobacterium *L. majuscula* had the highest NO_3_^−^ and PO_4_^3−^ uptake rates (Fig. 5). *Dichothrix* spp., which is also a filamentous cyanobacterium, showed lower uptake rates than *L. majuscula*, but its tightly clumped morphology may cause nutrient competition among neighboring thalli resulting in lower overall nutrient uptake rates^30^. We note that, in addition to a high surface:volume ratio, other species traits such as nutrient storage capacity^10^,^15^ and efficiency of internal nutrient recycling^32^ will also affect nutrient uptake rates, but these aspects were not investigated in the present study.

The NH_4_^+^ uptake kinetics of most species followed a biphasic pattern, whereby rapid initial ‘surge uptake’ was followed by slower, concentration-dependent uptake. Interestingly, the high nutrient concentrations offered in our study did not saturate nutrient uptake rates of the investigated species (i.e., the maximum uptake capability was not reached). Surge uptake is often observed when N-starved algae are offered a NH_4_^+^ pulse^15^,^33^,^34^,^35^, and likely represents the rapid reloading of depleted intracellular nitrogen pools^36^. At the pH of seawater (pH 8.1), 7% of the total ammonium-N is available in the form of NH_3_ (i.e., the pK_a_ of NH_3_ ≈ 9.25). Since plasma membranes are permeable for NH_3_ but not for NH_4_^+^ ions, passive diffusion of NH_3_ over the plasma membrane likely underlies the observed initial surge uptake. Subsequent NH_4_^+^ uptake rates of most species showed positive linear relationships with ambient NH_4_^+^ concentrations, supporting other studies reporting linear uptake kinetics for NH_4_^+ ^15,37,38. Most macroalgae possess active NH_4_^+^ transporters^36^. Therefore, it is likely that the observed linear uptake kinetics results from a combination of active NH_4_^+^ transport (with saturating enzyme kinetics) and passive NH_3_ diffusion.

Another explanation for the observed linear uptake kinetics could lie in the fact that the experimental NH_4_^+^ concentrations (0–50 μM) were too low to induce a saturating response. This is consistent with several studies from temperate waters in which nutrient uptake was saturated only when the incubation water was enriched by more than 50 μM NH_4_^+ ^34,39, although nutrient saturation at concentrations lower than 50 μM NH_4_^+^ has occasionally been found for temperate macroalgae^40^. Regardless, at NH_4_^+^ concentrations of 5 μM (as observed in the sediment plume), the NH_4_^+^ uptake rates of all species investigated in this study were still well below their maximum uptake capability. Therefore, sediment plumes with even higher NH_4_^+^ concentrations are likely to further fuel macroalgae, turf algae and cyanobacteria, increasing their ability to grow and overtake space within reef communities at the expense of live coral^7^.

Turf algae and the cyanobacteria *Dichothrix* spp. and *L. majuscula* showed initial PO_4_^3−^ surge uptake, but macroalgae and *M. mirabilis* did not. We are not aware of other studies reporting PO_4_^3−^ surge uptake by cyanobacteria and turf algae, but surge uptake has been observed in P-deficient macroalgae^41^,^42^. It has been hypothesized that this initial high uptake rate results from diffusion of PO_4_^3−^ into the organism’s extracellular space, a process that may take only a few minutes and mistakenly regarded as surge uptake^36^,^41^. Turf algae and the cyanobacteria *Dichothrix* spp. and *L. majuscula* showed strong linear increases of their PO_4_^3−^ uptake rates with increasing ambient PO_4_^3−^ concentrations. Half-saturation constants for PO_4_^3−^ uptake by benthic algae are typically in the range of 0.5–15 μM of PO_4_^3−^ depending on species^39^,^43^ indicating that the PO_4_^3−^ concentrations in our experiments (0–1.5 μM) and measured in the sediment plume (~1 μM) were not high enough to saturate the P uptake rates of benthic algae. The combination of initial surge uptake and subsequent non-saturating linear PO_4_^3−^ uptake across the investigated concentration range, indicate that turf algae and benthic cyanobacteria can rapidly respond to the temporarily enhanced PO_4_^3−^ concentrations in passing sediment plumes.

Of all reef organisms considered here, the highest nutrient uptake rates per unit biomass were found for turf algae followed by benthic cyanobacteria (i.e., *L. majuscula*) and *Cladophora* spp., which are all species capable of forming algal blooms^4^,^24^,^25^,^44^,^45^. The ability of these species to quickly capitalize on newly available nutrients explains their capacity to proliferate uncontrollably in regularly eutrophied coastal waters^5^,^24^. Like many other reefs, our study site has experienced a shift from a coral-dominated community about 40 years ago to a present-day reef community dominated by turf algae and macroalgae^46^,^47^,^48^. We used our data to calculate the distribution of nutrients supplied by sediment plumes over the different species. These model calculations are based on several simplifying assumptions, for instance nutrient concentrations experienced by benthic reef organisms during a run-off event may differ from nutrient concentrations in the overlying sediment plume, uptake kinetics in the field may differ from uptake rates determined under laboratory conditions, we ignored nutrient losses by the organisms (e.g., excretion of organic nutrients), uptake data were obtained for only one coral species, and some functional groups (e.g., sponges) were not investigated. Furthermore, the enhanced turbidity and sedimentation rates associated with sediment plumes^49^,^50^ and spatio-temporal variation in water velocity^51^,^52^ are also known to affect nutrient uptake rates. Hence, our simple model calculations should be interpreted with caution and provide at best a first approximation. Nevertheless, the results indicate that turf algae (at 5 m depth) and opportunistic macroalgae such as *L. variegata* and *Dictyota* spp. (at 20 m depth) were able to acquire a disproportionally large fraction of nutrients from episodic runoff events, which may help to explain their competitive success relative to the historically abundant coral species.

We urge for future studies investigating the effects of terrestrial runoff after heavy rainfall in further detail, as our results indicate that these episodic events can have major impacts on the nutrient budgets and competitive relationships in reef ecosystems.

## Methods

### Research site and community composition

**Figure 8:**
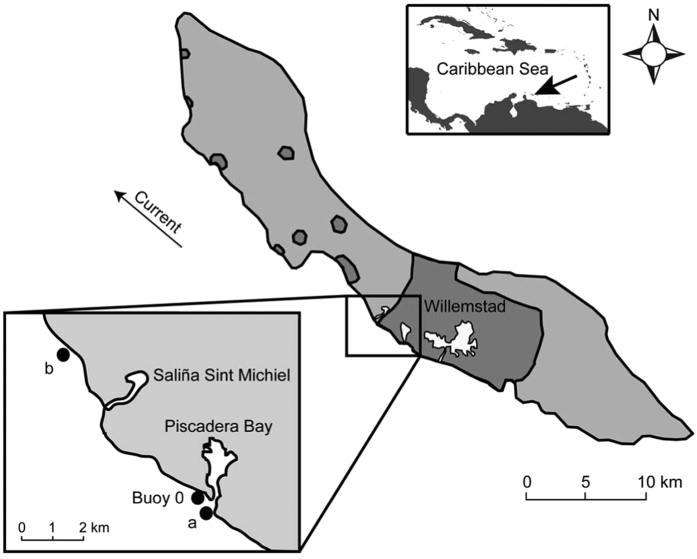
Map of the island of Curaçao, Southern Caribbean. Shading indicates urban areas (dark grey zones). The inset shows the research site Buoy 0 and the locations of the sediment plumes observed at Piscadera Bay (**a**) and Pest Bay (**b**) (see photographs in Fig. 1). Adapted with permission from Den Haan *et al.*^46^.

This study was conducted during the spring (March-June) of 2012 and 2013 at the fringing reef of research site ‘Buoy 0’ on the leeward side of the island of Curaçao, Southern Caribbean (12°7′29.07″N, 68°58′22.92″W; Fig. 8). The research site is located ~500 m from the opening of Piscadera Bay (12°7′21.42″N, 68°58′12.52″W), a semi-enclosed and highly eutrophied bay receiving nutrients from coastal residencies and the emergency overflow of a sewage treatment plant^53^. During the course of our study, we observed rainfall at Buoy 0 approximately every second day, both in 2012 and 2013 (average rainfall was 4.17 ± 0.61 and 4.64 ± 0.70 mm 24 h^−1^ (±s.e.m.), respectively). Benthic community composition at Buoy 0 was estimated from photoquadrat surveys that were conducted at 5 and 20 m depth in a parallel study by Den Haan *et al.*^46^. In shallow water (5 m depth), the benthic community at Buoy 0 consisted foremost of turf algae (cover: 44%) and white patches of sand (28%), interspersed by benthic cyanobacterial mats (11%), hard corals (7%) and macroalgae (6%; mainly *Dictyota menstrualis*). *M. mirabilis* was the most abundant coral species in these shallow waters, with a total cover of 2.5%. The cyanobacterial mats^54^,^55^ were dominated by *Oscillatoria*, *Hydrocoleum* and *Lyngbya* spp.; we did not observe mats of benthic eukaryotic algae at our study site. In deeper water (20 m depth), the benthic community consisted of macroalgae (43%; mainly *L. variegata* and *D. pulchella*), hard corals (17%, including 1.3% *M. mirabilis*), white sand (12%), turf algae (10%) and cyanobacterial mats (9%).

The standing biomass (in g DW per m^2^ of reef area) of the aforementioned species on the reef was calculated by multiplying its percent cover on the reef (i.e., from Den Haan *et al.*^46^) with its dry weight per unit of area, the ‘areal density’ (not to be confused with population density in terms of n m^−2^). The areal density of each benthic taxon was defined as its dry weight per m^2^ of bottom surface covered by that species, taking into account the 3-D structure of both the reef and the organisms on it. To produce areal density estimates for each macroalga, we photographed 25 quadrats (0.25 m^2^) over differently sized patches of *H. opuntia*, *Dictyota* spp. and *L. variegata*. The macroalgae were then collected from the quadrats, manually cleaned from epiphytes and detritus, and dried at 60 °C for at least 3 days to determine their dry weight. After quantification of their cover inside the quadrats using ImageJ^56^, a conversion factor relating the algae’s dry weight to their cover inside the photo-quadrats could be calculated. A similar conversion factor was derived for turf algae by cutting 35 strips from plastic bottles incubated at 5 and 20 m depth for six weeks (for details on how we collected turf algae, see paragraph ‘Collection of benthic organisms’ below). Each strip was photographed to measure turf algal cover (similar as above) after which turf algae were scraped off the plastic and freeze-dried in a Scanvac CoolSafe Freeze-dryer (Scala Scientific B.V., Ede, The Netherlands) to determine their dry weight. Following Jantzen *et al.*^57^ we multiplied the dry weight of turf algae with 1.5 to take into account the fact that the actual reef surface is topographically more complex than the plastic strips from which we sampled turf algae. Cyanobacterial mats and attached sediment were collected at 5 and 20 m depth from 10 small quadrats of 0.01 or 0.04 m^2^ placed on sandy sediment with 100% cyanobacterial cover, using a 50 ml Terumo syringe (Terumo Europe, Leuven, Belgium). The cyanobacteria were freeze-dried using a Scanvac CoolSafe Freeze-dryer and combusted at 450 °C for 4 h using an Air Recirculating Chamber Furnace (Carbolite, Hope Valley, UK) so that their areal density could be calculated from the difference in weight between the original sample (cyanobacteria with sediment particles) and combusted sample (only sediment particles). Most cyanobacterial mats at Buoy 0 were located on flat sandy sediments with a simple 2D structure. To estimate the areal density of hard corals, we first estimated the percent cover of all major coral species at 5 and 20 m depth using CPCe^58^ as described in the previous paragraph. For each coral species identified, we multiplied its percent cover by a 2-D to 3-D conversion factor (Table 2 in Holmes^59^) depending on the morphology of the coral species of interest to obtain the surface area per m^2^ reef for each coral species. Subsequently, we multiplied these data with a species-specific surface area to biomass conversion factor provided by Hardt^60^ (see Table 5.1 in Hardt^60^) to obtain the biomass per m^2^ reef for each species. Summing up the biomasses of all major coral species at Buoy 0 resulted into the total coral biomass per m^2^ reef. Finally, to obtain the areal density of all hard corals together, we divided the total coral biomass per m^2^ reef by the total percent cover of all corals on the reef.

### Nutrient analysis

Water was collected from 5, 10 and 20 m depth, at ~10 cm above the reef slope, using 5.3 L Plexiglas tubes. Because previous measurements^46^ showed that dissolved nutrient concentrations at Buoy 0 were homogeneous over the upper 20 m of the water column, we mixed the water collected from 5, 10 and 20 m depth. Subsequently, three water samples were taken from the mixed water (n = 3) using a 50 ml Terumo syringe (Terumo Europe, Leuven, Belgium) for nutrient analysis. We repeated this procedure every second day from October 31^st^ till December 10^th^, 2011 (n = 10) to obtain the average ambient NH_4_^+^, NO_3_^−^ and PO_4_^3−^ concentrations in seawater. Sediment plume water originating from the nearby Piscadera Bay (Fig. 8) was sampled on 23 November 2011. Using a 50 ml Terumo syringe (Terumo Europe, Leuven, Belgium) we extracted six water samples within the plume at approximately 1 m depth (n = 6). The water samples were immediately filtered using 0.22 μm Acrodisc filters and stored in 6 ml polyethylene vials (PerkinElmer, MA, USA) at −20 °C until analysis. Concentrations of NH_4_^+ ^61, NO_3_^− ^62 and PO_4_^3− ^63 were analyzed using a QuAAtro continuous flow auto-analyzer (Seal Analytical, UK) at the Royal Netherlands Institute for Sea Research (Texel, The Netherlands).

### Collection of benthic organisms

Benthic organisms dominating the community at Buoy 0 were collected at 5 and 20 m depth to determine their nutrient uptake rates. We selected one abundant coral species (*M. mirabilis*; branches ~5 cm in length), six macroalgal species (*Cladophora* spp. (only at 5 m depth), *D. marginata*, *D. menstrualis* (only at 5 m depth), *D. pulchella* (only at 20 m depth), *H. opuntia*, *L. variegata* (only at 20 m depth)), two benthic cyanobacteria (*Dichothrix* spp. (only at 5 m depth) and *L. majuscula*) and turf algae. Turf algae were not collected directly from the reef, because scraping them off the rocky surface would damage their tissue. Turf algae were instead grown on the exterior of 1.5 L square plastic bottles (FIJI Water Company, CA, USA), which were placed inside 1 m^3^ chicken-wired cages (mesh size = 2.5 cm) to minimize grazing by large herbivores. The caged bottles were placed at 5 and 20 m depth, about 0.5 m above the reef to avoid overgrowth by benthic communities already present. After 6 weeks the bottles were covered by turf algal communities comprising all major taxa of natural turf communities observed on the reef, including Chlorophyta, Rhodophyta, Phaeophyceae and Cyanobacteria^64^. The turf algae were subsequently collected by cutting out plastic strips (±25 cm^2^) from these turf algal-covered bottles.

After collection, benthic organisms were gently cleaned from epiphytes and detritus using tweezers and transported to the lab in darkened plastic Ziploc bags within a cool box filled with ambient seawater (27–29 °C). Within one hour of collection, the organisms were used for nutrient uptake experiments, with the exception of *M. mirabilis*. This coral species was collected one day earlier and placed in a flow-through aquarium system, where the corals were provided with seawater of 27–29 °C that was directly pumped from the reef, and light levels of ~100 μmol photons m^**−**2^ s^**−**1^ during the daytime, using the same setup as in Den Haan *et al.*^46^. We ensured the *M. mirabilis* polyps had at least 24 hrs to recover from sampling. After 24 hrs the polyps were always fully extended, and we started the nutrient uptake experiments.

### Nutrient uptake experiments

In a series of laboratory experiments, we measured the nutrient uptake rates of all collected organisms in response to a nutrient pulse. The species were incubated in 300 ml glass jars that were acid-washed (10% HCl) prior to usage and filled with filtered natural seawater (0.22 μm Whatman cellulose acetate membrane filters). The average biomass (g dry weight, ±s.e.m.) per glass jar was as follows: *M. mirabilis* 7.61 ± 0.39; turf algae 0.20 ± 0.01; *H. opuntia* 5.78 ± 0.37; *Dictyota* spp. 0.46 ± 0.06; *L. variegata* 0.39 ± 0.01; *Cladophora* spp. 0.24 ± 0.02; *D. marginata* 0.34 ± 0.01; *Dichothrix* spp. 0.48 ± 0.05; *L. majuscula* 0.31 ± 0.02. Because *H. opuntia* is a calcifying algal species, its dry weight is higher than that of other benthic algae. Ideally we would have mimicked the nutrient concentrations that we found in the sediment plume on the reef (Fig. 2). However, at the time we started the laboratory experiments we did not yet have the nutrient data from the sediment plume. Therefore, we chose the following five nutrient treatments (end-concentrations) based on earlier eutrophication experiments on reefs^20^,^24^,^25^: (1) low NH_4_^+^ (5 μM) and (2) high NH_4_^+^ (50 μM) treatment by adding NH_4_Cl to the filtered natural seawater; (3) high NO_3_^−^ (25 μM) treatment by adding NaNO_3_; (4) low PO_4_^3−^ (0.88 μM) and (5) high PO_4_^3−^ (1.75 μM) treatment by adding KH_2_PO_4_. These nutrient concentrations reflect, at least in order of magnitude, natural increases in nutrient concentrations during runoff events (e.g., Lapointe *et al.*^22^ and Costa *et al.*^23^ with references therein).

For each nutrient treatment, a nutrient stock (i.e., one of the five aforementioned nutrient treatments) was created in a distinct 25-liter plastic container on the same day a nutrient uptake experiment was conducted. For each species and each nutrient treatment, we used ten glass jars. Each time we performed the nutrient uptake experiment we used only one species that originated either from 5 or 20 m depth, and applied only one of the five nutrient treatments (i.e., we conducted one single nutrient uptake experiment per day). We placed the species to be investigated in 9 of the 10 glass jars (n = 9 per species per nutrient treatment), and the tenth jar served as a control to ensure that nutrient concentrations remained constant in absence of benthic organisms. For experiments involving turf algae, a clean plastic strip was added to the control glass to check if the plastic on which turf algae were propagated affected nutrient concentrations inside the jar. As nutrient concentrations in all controls remained constant, observed changes in nutrient concentration during the experiments were attributed to the organisms placed in the jars.

The ten glass jars were placed in a large aquarium (80 × 40 × 20 cm). The water level in the aquarium was too low to enter the jars, but the circulating fresh seawater ensured that all samples experienced temperatures similar to those on the reef (27–29 °C). Each jar was individually aerated using Vibra-Flo 2 or 3 aquarium air pumps (Blue Ribbon Pet Products, NY, USA) equipped with 0.22 μm Acrodisc filters to minimize potential aerial contamination. Gentle bubbling of the water ensured constant water movement inside the jars throughout the uptake experiment. Ten 50 W spotlights provided a constant light intensity of 200 μmol photons m^−2^ s^−1^, as measured using a LI-1000 Datalogger (LI-COR, Lincoln, Nebraska, USA), which is similar to the light conditions measured at our study site at 20 m depth during sunny days^65^. This light availability was sufficient to provide (almost) saturating light conditions for all species in our study, except *M. mirabilis*, according to photosynthesis-irradiances curves (n = 5) made *in situ*^65^. Hence, the ten light spots used during the nutrient uptake experiments in the laboratory should have provided ample light for most organisms collected at 5 and 20 m depth. The coral *M. mirabilis* was an exception, because its photosynthetic rate^65^ still increased almost linearly with irradiance at 200 μmol photons m^−2^ s^−1^ and only started to approach saturating values at high irradiance levels above 800 μmol photons m^−2^ s^−1^.

Uptake rates of NH_4_^+^ and PO_4_^3−^ were determined from the decrease in NH_4_^+^ and PO_4_^3−^ concentrations in each jar over time. This so-called ‘drawdown method’ is an easy and well-established method for macroalgae^15^,^35^,^38^,^66^, corals^67^,^68^, and even microalgae^69^, with the advantage that the uptake rate can be quickly determined at different external nutrient concentrations. The first water sample was taken before an organism was placed inside the jar. After adding the organism, 5 ml water samples were taken at specific time intervals. In the NH_4_^+^ uptake experiments, water samples were taken after 10, 20, 30, 60 and 120 min. In the PO_4_^3−^ uptake experiments, water samples were taken after 10, 20, 30, 40, 50 and 60 min, at which point the PO_4_^3−^ detection limit was often reached. After a water sample was taken during a NH_4_^+^ or PO_4_^3−^ uptake experiment, it was immediately filtered through 0.22 μm sterile Acrodisc filters (Pall Corporation, NY, USA) into 6 ml Polyethylene vials (PerkinElmer, MA, USA). Water samples were immediately analyzed for NH_4_^+^ and PO_4_^3−^ according to Holmes *et al.*^70^ and Murphy & Riley^63^, respectively, using a T60 Visual Spectrophotometer (PG Instruments Ltd, Wibtoft, UK). At the end of the incubation experiment, the algae and cyanobacteria were quickly rinsed with distilled water and stored in pre-weighed aluminum foil at −20 °C. The samples were freeze-dried overnight in a Scanvac CoolSafe Freeze-dryer (Scala Scientific B.V., Ede, The Netherlands) to determine their dry weight. The total dry weight of all live tissues of *M. mirabilis* was approximated from its surface area according to Hardt^60^. The maximum NH_4_^+^ and PO_4_^3−^ uptake rate (V_max_) of each species was calculated from its total uptake during the first 10 min (i.e., t_0_ − t_10_), and expressed in μmol g^−1^ DW^−1^ h^−1^. The V_max_ was calculated only from nutrient uptake experiments that used a high initial nutrient treatment (i.e., 50 μM NH_4_^+^ or 1.75 μM PO_4_^3−^), as the highest initial nutrient concentration consistently resulted in the V_max_ for all species studied.

On Curaçao we did not have the opportunity to measure the uptake of NO_3_^−^ spectrophotometrically, because we lacked cadmium, titanium chloride and hydrazine for the required NO_3_^−^ reduction assays. Hence, the NO_3_^−^ uptake rate was determined from the incorporation of the stable isotope ^15^N over a 2 h incubation period. At the onset of each incubation, 25 μM Na^15^NO_3_ (98 at%, Sigma Aldrich, Zwijndrecht, The Netherlands) was added to nine glass jars with benthic organisms, whereas the tenth glass jar served as control by incubating the respective organism without addition of Na^15^NO_3_. The ^15^N concentrations of the control organisms were used as a reference for the organisms’ background ^15^N concentrations (i.e., control sample). After 2 hrs of incubation, the dry weight of the corals, algae and cyanobacteria were estimated similarly as during the NH_4_^+^ and PO_4_^3−^ uptake experiments (see above).

The tissue δ^15^N content of the samples were determined using a Thermofinnigan Delta Plus isotope ratio mass spectrometer (Bremen, Germany) connected to a Carlo Erba Instruments Flash 1112 Element Analyzer (Milan, Italy). Algae samples were ground to powder and transferred into tin capsules (size: 12 × 6 × 6 mm) that were folded into small pellets. In corals, zooxanthellae can assimilate NO_3_^−^ but the animal polyps cannot^71^,^72^. For analysis of the NO_3_^−^ uptake rate by zooxanthellae in the coral *M. mirabilis*, coral tissue was removed from the underlying skeleton using a toothbrush and suspended in a 15 ml test tube containing filtered seawater (Whatman GF/F). This suspension was centrifuged twice at 4000 rpm for 20 min in an EBA 21 Centrifuge (Hettich Laborapparate, Bäch, Germany), to concentrate zooxanthellae at the bottom of the tube. The zooxanthellae were pipetted out of the tube, filtered onto a Whatman GF/F filter that was pre-combusted at 450 °C for 4 hrs using an Air Recirculating Chamber Furnace (Carbolite, Hope Valley, UK), and stored at −20 °C for at least three days before being freeze-dried. For *M. mirabilis*, Whatman GF/F filters loaded with its zooxanthellae were directly packed into the tin capsule and folded into a small pellet. The powder (algae and cyanobacteria) or filter (zooxanthellae) within the tin capsule was weighed and its δ^15^N content (in ‰) was quantified as:


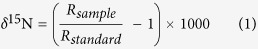


where *R*_*sample*_ is the isotope ratio ^15^N/^14^N of the sample and *R*_*standard*_ is the isotope ratio of atmospheric N_2_ (i.e., *R*_standard_ = 0.0036765). The δ^15^N measurements were calibrated against the laboratory standards urea (δ^15^N = −40.81‰) and acetanilide (δ^15^N = 1.3‰). The NO_3_^−^ uptake rate (in μmol N g^−1^ DW h^−1^) of each species was calculated as:


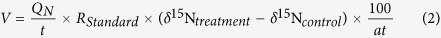


where *Q*_*N*_ is nitrogen content of the tissue (in mmol N g^−1^ DW), *t* is the incubation time of 2 hrs, δ^15^N_*treatment*_ is the δ^15^N of ^15^N-enriched samples, δ^15^N_*control*_ is the δ^15^N of the control sample, and *at* is the atomic concentration (at%) of ^15^N in the nitrate pulse we supplied. V_max_ for NO_3_^−^ was calculated by dividing V in equation 2 by two hours, and expressed in μmol g^−1^ DW^−1^ h^−1^.

NH_4_^+^ and PO_4_^3−^ uptake rates (V) were plotted as function of the nutrient concentrations in the medium. Since nutrient concentrations changed during the course of the uptake experiments, we chose to calculate moving averages of the nutrient concentrations using three consecutive time points to suppress measurement noise in the nutrient data (e.g., the nutrient concentration at t_20_ was calculated as ([t_10_] + [t_20_] + [t_30_]/3). If the uptake pattern of an organism was biphasic, the initial nutrient uptake rate (surge uptake) during the first 10 min of the experiment was excluded from this analysis. Furthermore, negative nutrient uptake rates (obtained when nutrient concentrations occasionally increased during the uptake experiments) were also excluded from the analysis.

### Distribution of nutrients among different species

The calculated species- and nutrient-specific uptake rates were used to estimate how nutrients delivered by episodic nutrient pulses were used by different members of the reef community. The uptake of nutrients by a coral reef community consisting of *n* species can be described by the following set of differential equations:


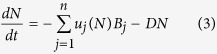



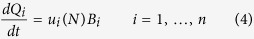


where *N* is the nutrient concentration in the nutrient plume, *Q*_*i*_ is the total amount of nutrient in species *i*, *u*_*i*_(*N*) is the biomass-specific nutrient uptake rate of species *i* as a function of the ambient nutrient concentration *N*, *B*_*i*_ is the biomass of species *i*, and *D* is the rate at which the nutrient concentration in the plume decreases through other processes (e.g., mixing with open ocean water, denitrification). Because it was not feasible to measure the nutrient uptake kinetics of all species, we assume that the nutrient uptake rate of *M. mirabilis* is representative for all hard corals at our study site. Similarly, we assume that the average uptake rates of *Dichothrix* spp. and *L. majuscula* represent the uptake rates of cyanobacterial mats at 5 m depth at our study site, and that *L. majuscula* represents the uptake rates of cyanobacterial mats at 20 m depth (i.e., *Dichothrix* spp. did not occur at 5 m depth at our study site).

If the nutrient uptake rates of the species depend linearly on ambient nutrient concentration (i.e., *u*_*i*_(*N*) = *a*_*i*_*N*), differential equation (3) can be solved, and the decrease of the ambient nutrient concentration through time can be written as:


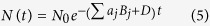


where *N*_*0*_ is the initial nutrient concentration in the nutrient plume. Nutrient acquisition by each species in the community is then obtained by inserting equation (5) into equation (4) and subsequent integration, which results in:


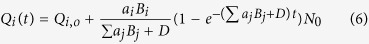


where *Q*_*i,*0_ is the initial amount of nutrients in the tissue of species *i*.

Equation (6) shows that each species acquires a fraction *a*_*i*_*B*_*i*_/(Σ*a*_*j*_*B*_*j*_ + *D*) of the nutrients delivered by the pulse. Hence, species with more biomass and/or higher nutrient uptake rates will acquire a larger fraction of the total nutrient input. The magnitude of parameter *D* is unknown for our research site. Therefore, we cannot accurately estimate which fraction of the nutrients from the sediment plume vanishes into the open ocean or is taken up by plankton. However, from the measured nutrient uptake rates and biomasses of the studied species we can calculate the fraction *a*_*i*_*B*_*i*_/Σ*a*_*j*_*B*_*j*_, indicative of the proportion of nutrients that are taken up by different members of the reef community at our research site.

### Statistical analyses

Two-sample Student’s *t*-tests (for equal variances) or Welch’s *t*-tests (for unequal variances) were used to test whether nutrient concentrations in normal oceanic water differed from those in sediment plumes after heavy rainfall.

We applied a two-way analysis of variance to test if maximum nutrient uptake rates (V_max_) differed among species and between depth-origin of the species (i.e., to study the effect of depth on V_max_, we only included those organisms that were found at both 5 and 20 m depth on the reef). The data were log-transformed if this improved homogeneity of variance, as tested by Levene’s test. Post-hoc comparisons of the means were based on Tukey’s HSD test using a significance level (α) of 0.05.

Nutrient uptake rates can follow a biphasic pattern, whereby nutrient uptake is highest immediately after a nutrient pulse, but subsequently shifts to a lower uptake rate^35^,^66^. We tested for the presence of such biphasic patterns by determining if nutrient uptake rates during the first 10 min of the experiments (time interval t_0–__10_) were significantly higher than uptake rates during the next 10 min (time interval t_10–__20_) using paired samples *t*-tests.

Linear regression was used to test whether nutrient uptake rates increased significantly with increasing ambient nutrient concentrations. Normality and homoscedasticity of the residuals in the regression analysis was assessed with the Shapiro-Wilk test (α = 0.05) and by visual inspection of the residuals, respectively. The Shapiro-Wilk test indicated that in nearly all cases the distribution of the residuals met the normality assumption. The only exception was the PO_4_^3−^ uptake rate of *L. majuscula*, where all data points except one were near the regression line (Fig. 6f). The normality test was passed when this data point was removed, but we decided to maintain this data point because linear regression tends to be quite robust against deviations from normality and there were no clear reasons to remove this particular point as an outlier. Visual inspection indicated that most residuals met the homoscedasticity assumption of linear regression, but the magnitude of the residuals of *M. mirabilis* and turf algae increased with the NH_4_^+^ concentration (Fig. 6a,b). We did not transform the NH_4_^+^ uptake rates of *M. mirabilis* and turf algae to improve homoscedasticity, however, because the other regressions in Fig. 6 and Supplementary Figs S5 and S6 did not show signs of heteroscedasticity and we preferred to apply the same statistical analysis to all species.

## Additional Information

**How to cite this article**: den Haan, J. *et al.* Nitrogen and phosphorus uptake rates of different species from a coral reef community after a nutrient pulse. *Sci. Rep.* **6**, 28821; doi: 10.1038/srep28821 (2016).

## Supplementary Material

Supplementary Information

## Figures and Tables

**Table 1:** Two-way analysis of variance of the maximum nutrient uptake rates, with species and depth as independent variables.

Effect	*df*_*1*_*, df*_*2*_	*F*	*P*
NH_4_^+^:			
** Species**	**8, 91**	**99.297**	**<0.001**
Depth	1, 91	2.085	0.152
** Species × Depth**	**3, 91**	**11.592**	**<0.001**
NO_3_^−^:			
** Species**	**6, 80**	**303.074**	**<0.001**
** Depth**	**1, 80**	**30.055**	**<0.001**
** Species × Depth**	**2, 80**	**14.105**	**<0.001**
PO_4_^3−^:			
** Species**	**8, 103**	**319.733**	**<0.001**
** Depth**	**1, 103**	**5.038**	**0.027**
** Species × Depth**	**4, 103**	**2.464**	**0.050**

Columns indicate the investigated effects, degrees of freedom (*df*_1_ and *df*_2_), the *F*-statistic (*F*_*df1,df2*_) and the corresponding probability (*P*). Significant results (*P* < 0.05) are indicated in bold.
