# Supplementary material for: Nitrogen and phosphorus uptake rates of different species from a coral reef community after a nutrient pulse
Source: Sci Rep. 2016 Jun 29;6:28821. doi: 10.1038/srep28821 (PMC4926277; doi:10.1038/srep28821)
Supplement: Supplementary Information [file srep28821-s1.pdf]

SUPPLEMENTARY MATERIALS

Nitrogen and phosphorus uptake rates of different species from a coral reef community after a nutrient pulse

Joost den Haan, Jef Huisman, Hannah J. Brocke, Henry Goehlich, Kelly R.W. Latijnhouwers, Seth van Heeringen, Saskia A.S. Honcoop, Tanja E. Bleyenberg, Stefan Schouten, Chiara Cerli, Leo Hoitinga, Mark J.A. Vermeij and Petra M. Visser

Supplementary figure S1

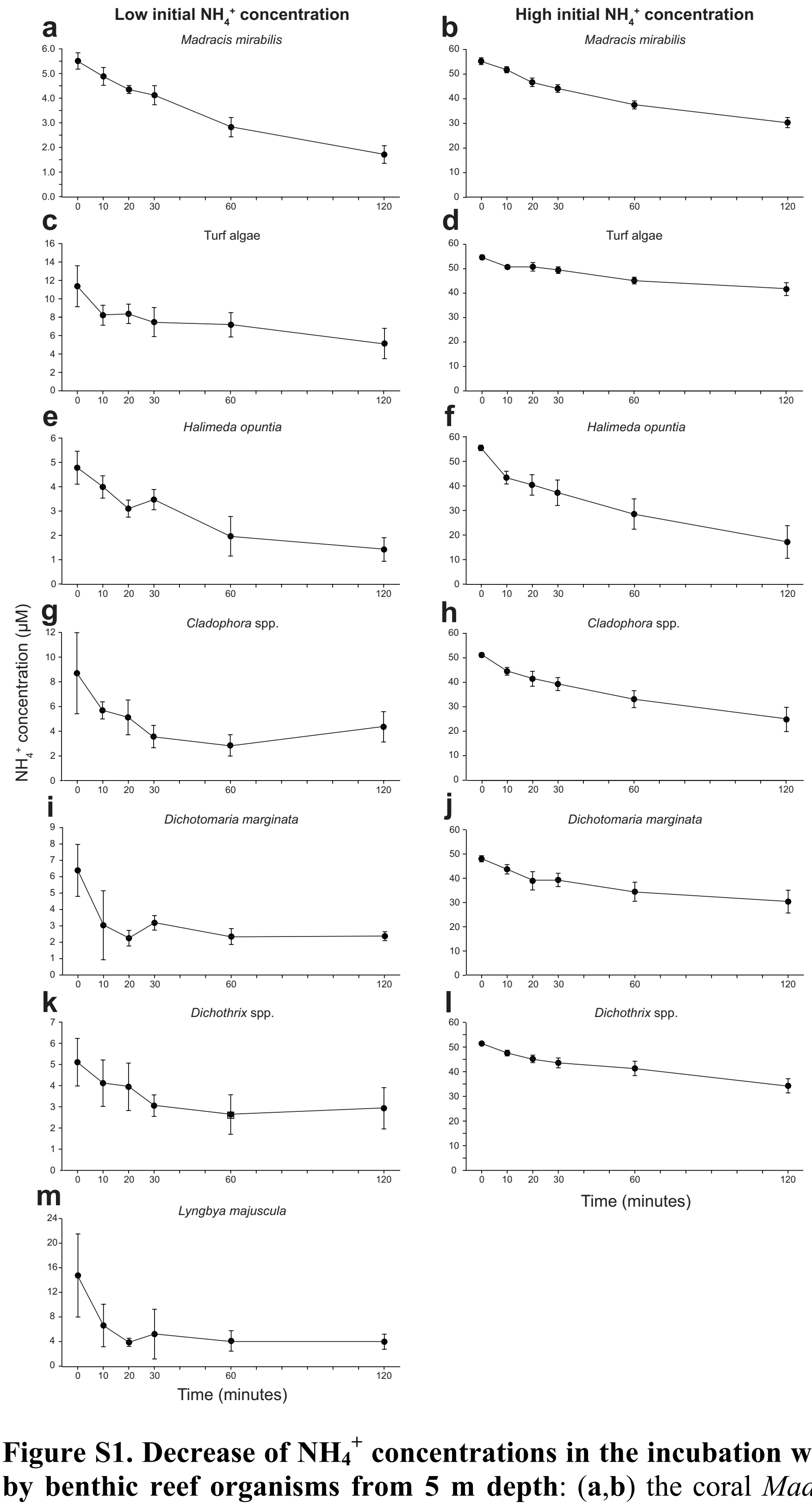

Figure S1. Decrease of NH_4^+ concentrations in the incubation water due to NH_4^+ uptake by benthic reef organisms from 5 m depth: (**a**,**b**) the coral *Madracis mirabilis*, (**c**,**d**) turf algae, (**e**,**f**) the macroalgae *Halimeda opuntia*, (**g**,**h**) *Cladophora* spp. and (**i**,**j**) *Dichotomaria marginata*, and (**k**,**l**) the benthic cyanobacteria *Dichothrix* spp., and (**m**) *Lyngbya majuscula*. The species were incubated at low (left column) and at high (right column) initial NH_4^+ concentrations. Error bars represent s.d. of the mean (n=9).

Supplementary figure S2

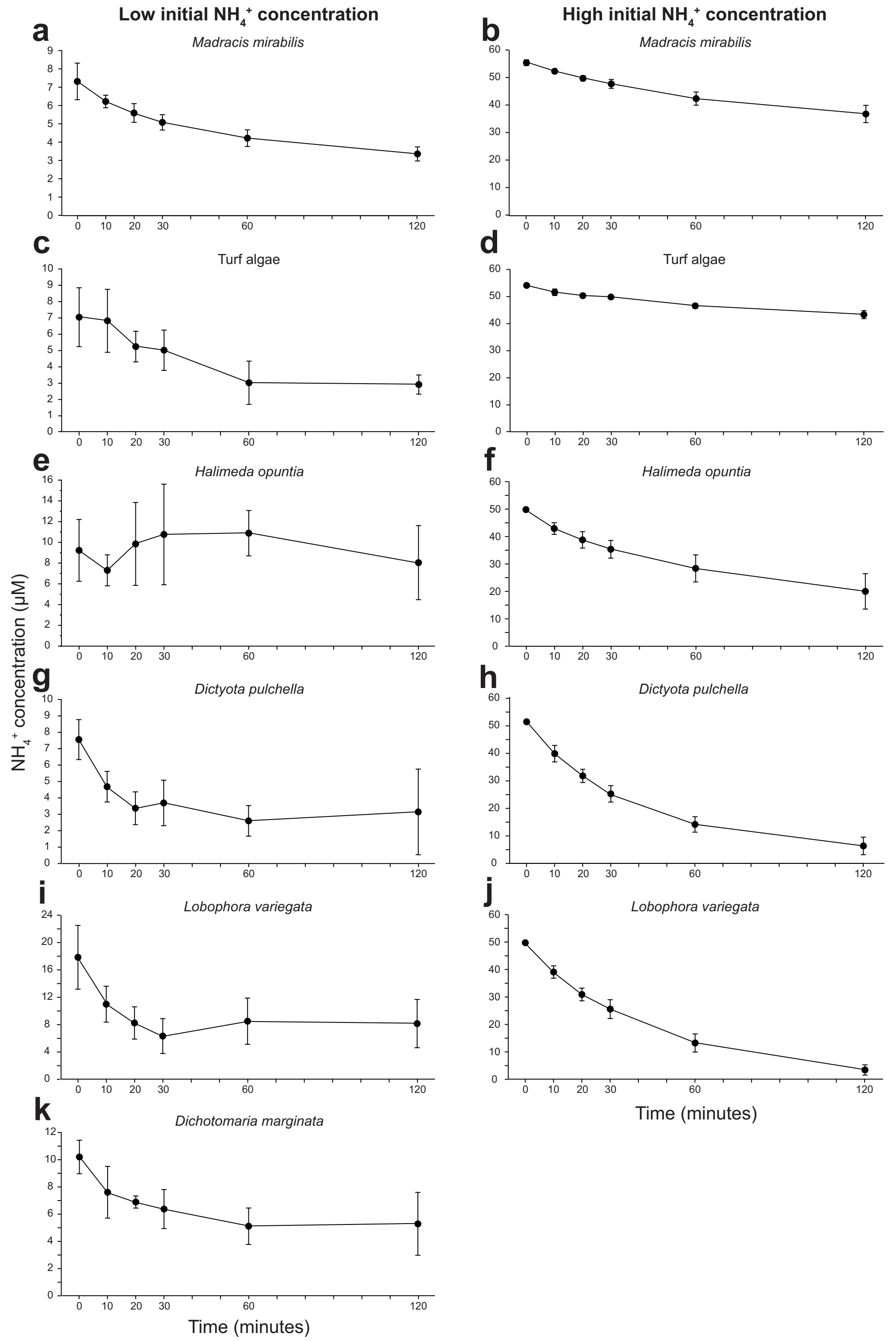

Figure S2. Decrease of NH_4^+ concentrations in the incubation water due to NH_4^+ uptake by benthic reef organisms from 20 m depth: (**a**,**b**) the coral *Madracis mirabilis*, (**c**,**d**) turf algae, (**e**,**f**) the macroalgae *Halimeda opuntia*, (**g**,**h**) *Dictyota pulchella*, (**i**,**j**) *Lobophora variegata*, and (**k**) *Dichotomaria marginata*. The species were incubated at low (left column) and at high (right column) initial NH_4^+ concentrations. Error bars represent s.d. of the mean (n=9).

Supplementary figure S3

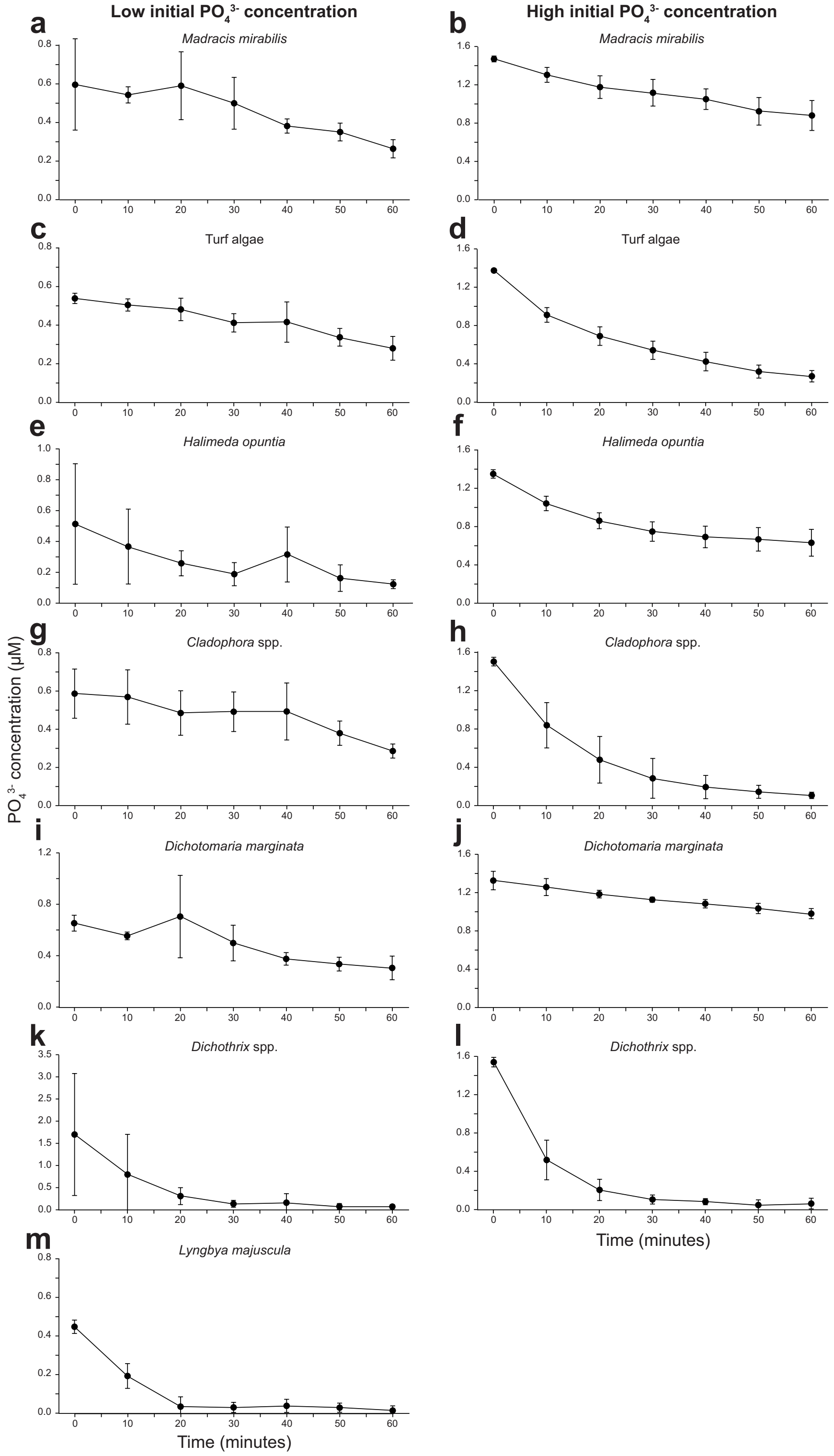

Figure S3. Decrease of PO_4^{3-} concentrations in the incubation water due to PO_4^{3-} uptake by benthic reef organisms from 5 m depth: (**a**,**b**) the coral *Madracis mirabilis*, (**c**,**d**) turf algae, (**e**,**f**) the macroalgae *Halimeda opuntia*, (**g**,**h**) *Cladophora* spp. and (**i**,**j**) *Dichotomaria marginata*, and (**k**,**l**) the benthic cyanobacteria *Dichothrix* spp., and (**m**) *Lyngbya majuscula*. The species were incubated at low (left column) and at high (right column) initial PO_4^{3-} concentrations. Error bars represent s.d. of the mean (n=9).

Supplementary figure S4

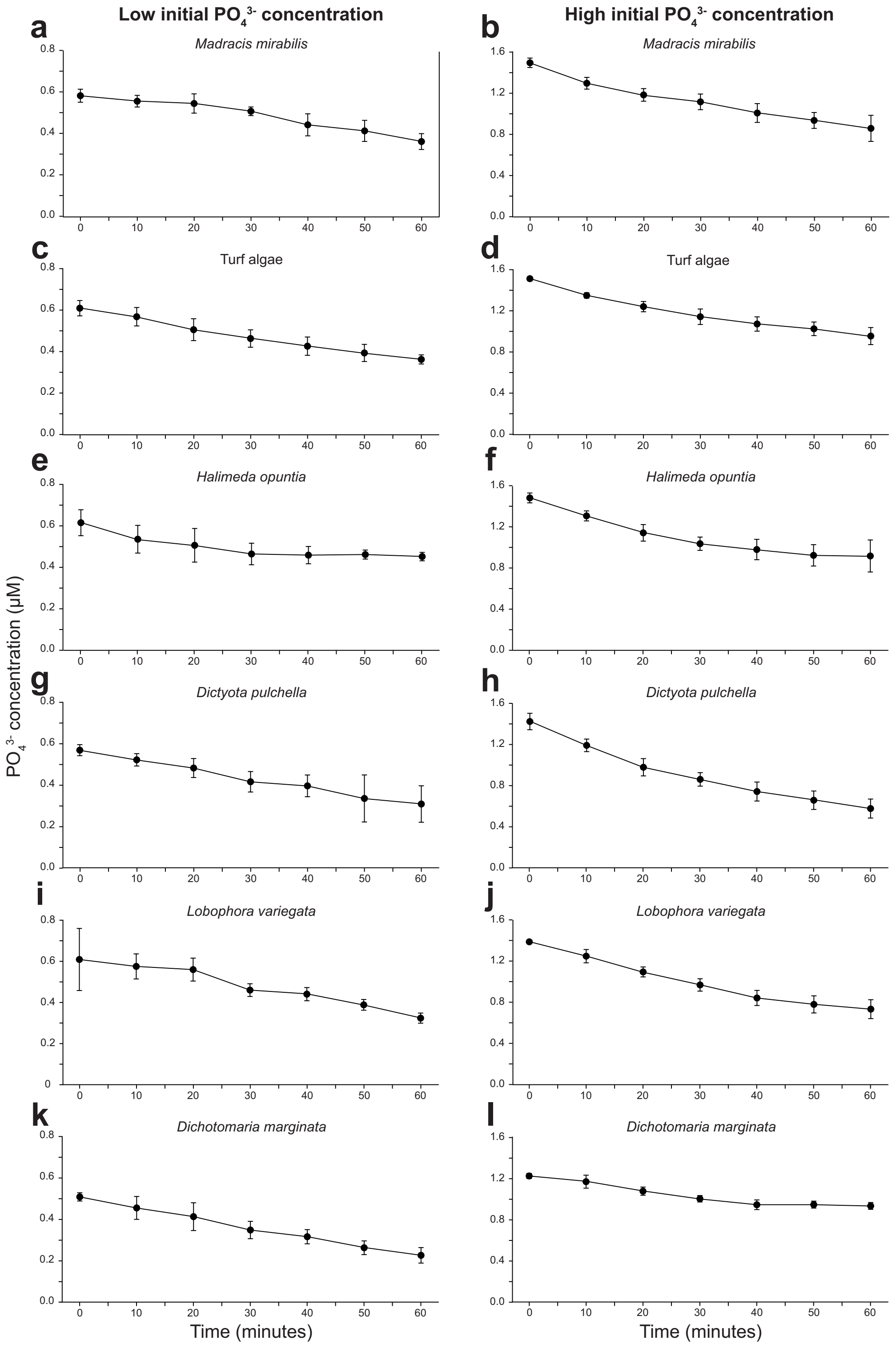

Figure S4. Decrease of PO_4^{3-} concentrations in the incubation water due to PO_4^{3-} uptake by benthic reef organisms from 20 m depth: (**a**,**b**) the coral *Madracis mirabilis*, (**c**,**d**) turf algae, (**e**,**f**) the macroalgae *Halimeda opuntia*, (**g**,**h**) *Dictyota pulchella*, (**i**,**j**) *Lobophora variegata*, and (**k,l**) *Dichotomaria marginata*. The species were incubated at low (left column) and at high (right column) initial PO_4^{3-} concentrations. Error bars represent s.d. of the mean (n=9).

Supplementary figure S5

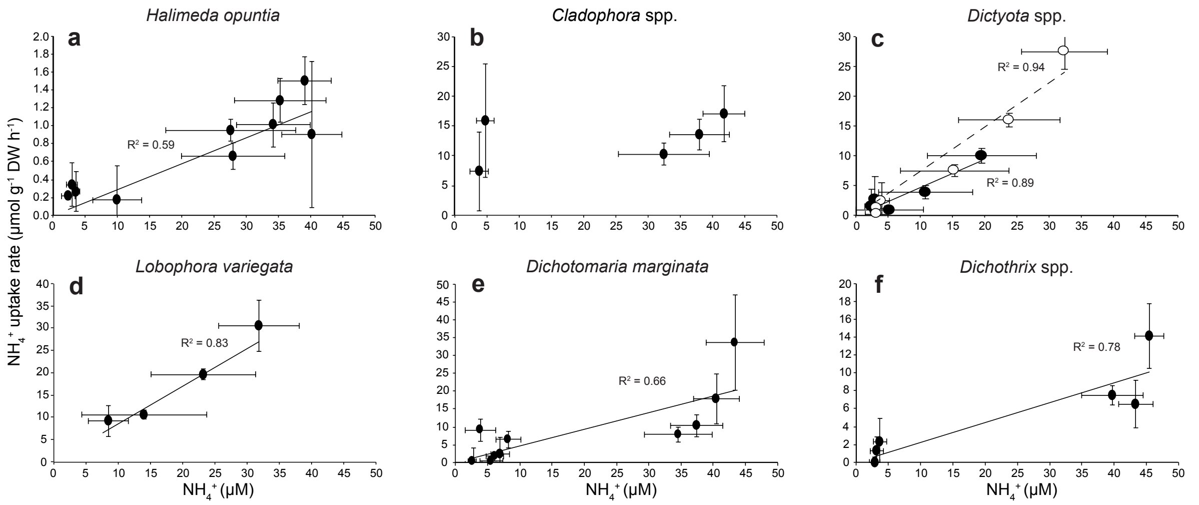

Figure S5. NH_4^+ uptake rates of the different species as function of the external NH_4^+ concentration. NH_4^+ uptake rates were calculated over 20 min time intervals during the nutrient uptake experiments. For those species with a biphasic NH_4^+ uptake, the first 10 min of the uptake experiments were not included. Data included specimens collected from both 5 and 20 m depth, and from uptake experiments performed at both low and high initial NH_4^+ concentrations. In (**c**), data for both *Dictyota menstrualis* (closed circles with solid trend line) and *Dictyota pulchella* (open circles with dashed trend line) are shown. Trend lines are based on linear regression forced through the origin, and are only shown if the regression was significant (P<0.05). Error bars represent s.d. of the mean (n=9)

Supplementary figure S6

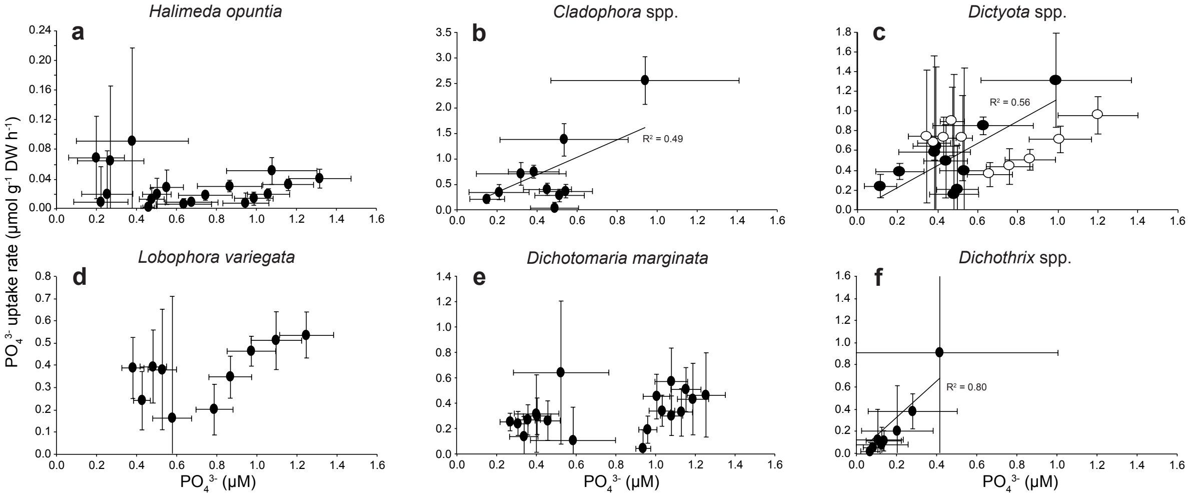

Figure S6. PO_4^{3-} uptake rates of the different species as function of the external PO_4^{3-} concentration. PO_4^{3-} uptake rates were calculated over 20 min time intervals during the nutrient uptake experiments. For those species with a biphasic PO_4^{3-} uptake, the first 10 min of the uptake experiments were not included. Data included specimens collected from both 5 and 20 m depth, and uptake experiments performed at both low and high initial PO_4^{3-} concentrations. In (**c**), data for both *Dictyota menstrualis* (closed circles with solid trend line) and *Dictyota pulchella* (open circles) are shown. Trend lines are based on linear regression forced through the origin, and are only shown if the regression was significant ($P<0.05$). Error bars represent s.d. of the mean (n=9)

Supplementary Table S1

Table S1. Comparison of nutrient uptake rates (for NH_4^+ and PO_4^{3-}) between the initial time interval t_0-t_{10} and the subsequent time interval t_{10}-t_{20} using paired sample *t*-tests. For each species, nutrient uptake rates were obtained from 9 replicate time series at 5 and/or 20 m depth and for high and low nutrient concentration treatments. Significant results (P<0.05) are indicated in bold.

	NH_4^+			PO_4^{3-}		
Species	*t*	*df*	*P*	*t*	*df*	*P*
Turf algae	**3.743**	**33**	**<0.001**	**3.176**	**31**	**0.003**
Lobophora variegata	**3.320**	**16**	**0.004**	0.102	15	0.920
Cladophora spp.	**4.075**	**16**	**<0.001**	1.324	17	0.203
Dichotomaria marginata	1.619	26	0.118	0.921	35	0.363
Halimeda opuntia	**4.869**	**33**	**<0.001**	2.015	31	0.053
Dictyota menstrualis	**2.954**	**16**	**0.009**	1.007	15	0.330
Dictyota pulchella	**2.408**	**16**	**0.028**	1.889	17	0.076
Lyngbya majuscula	**5.418**	**25**	**<0.001**	**4.972**	**26**	**<0.001**
Dichothrix spp.	**2.502**	**16**	**0.024**	**2.322**	**17**	**0.033**
Madracis mirabilis	-0.892	33	0.379	0.605	35	0.549

Supplementary Table S2

Table S2. Areal density (i.e., dry weight per unit of area) of the different benthic species. The areal densities assume that the 1 m^2 reef area is completely covered by the benthic organism (100%) and takes the 3-D structure of the reef and organism into account. ± represents s.e.m.

**Benthic species**	**Depth (m)**	**Areal density (g DW m^{-2})**
Hard corals	5	3065
	20	1961
Turf algae	5	127 ± 13.9
	20	68 ± 3.4
Halimeda opuntia	5	2590 ± 12.1
	20	2590 ± 12.1
Dictyota menstrualis	5	70 ± 0.9
Dictyota pulchella	20	70 ± 0.9
Lobophora variegata	20	100 ± 1.1
Cyanobacterial mats	5	11.1 ± 1.9
	20	6.2 ± 1.4
